# Effectiveness and cost-effectiveness of general health checks in adults: A systematic review

**DOI:** 10.1186/s12889-026-28436-4

**Published:** 2026-07-08

**Authors:** Yu Wang, Qin Zhao, Xin Zhu, Liang Wang, Linghui Xiang, Qing Liu, Tubao Yang, Jia Liu

**Affiliations:** 1 Xiangya School of Nursing, Central South University, Changsha, Hunan China; 2 Department of Nursing, Fudan University Shanghai Cancer Center, Shanghai, China; 3 Xiangya School of Public Health, Central South University, Changsha, Hunan China; 4 Department of Pharmacy, The Third Xiangya Hospital, Central South University, Changsha, Hunan China; 5 Health Management Medicine Center, The Third Xiangya Hospital, Central South University, Changsha, Hunan China; 6 Nursing Department, The Third Xiangya Hospital, Central South University, Changsha, Hunan China

**Keywords:** General health checks, Primary care, Preventive services, Cost-effectiveness, Systematic review

## Abstract

**Introduction:**

General health checks (GHCs) are widely used for disease prevention, however, their clinical effectiveness, psychological impact, and economic value remain debated. This study systematically evaluated the effectiveness and cost-effectiveness of GHCs in adults to update and expand upon existing reviews.

**Methods:**

We searched PubMed, Embase, Cochrane Library, Web of Science, CINAHL, SinoMed, CNKI, and WanFang from inception to July 1, 2025. Three reviewers independently screened titles and abstracts, and two reviewers assessed full-text eligibility, with disagreements resolved through discussion or consultation with a senior reviewer. We included randomized controlled trials, nonrandomized controlled trials, observational studies with control groups, mixed-methods studies, and economic evaluations comparing GHCs with usual care in adults. Risk of bias was assessed using Cochrane RoB 2.0, ROBINS-I, and other validated tools. The certainty of evidence was evaluated using the GRADE approach. Due to substantial heterogeneity, findings were synthesized narratively.

**Results:**

We included 74 articles representing 56 unique studies (22 RCTs, 5 NRCTs, 25 observational studies, 3 economic evaluations, 1 mixed-methods study), involving more than 17 million participants. RCTs generally did not show a significant reduction in all-cause mortality (76.9% of RCTs) or cardiovascular events (100% of RCTs) compared with usual care, whereas observational studies (6 of 8 studies) and analyses of high-risk subgroups suggested possible protective associations. GHCs were associated with increased detection of chronic diseases, greater uptake of preventive services, and modest improvements in patient-reported outcomes. Although GHCs modestly improved diet and physical activity, most trials (87.5%) reported little or no effect on smoking or hazardous alcohol use. Economically, GHCs reduced short-term healthcare costs and high-cost service utilization, but long-term cost-effectiveness remained uncertain (ICERs ranging from £900 to > AU$133,000 per QALY). Psychological effects were mostly neutral or showed small improvements, although increased suicide risk was reported in older adults in one large cohort.

**Conclusions:**

Current evidence suggests that GHCs may not substantially reduce mortality or cardiovascular events in the general population. However, they may be associated with earlier disease detection, modest risk-factor improvements, greater use of preventive care, and small psychological benefits. The long-term economic value remains uncertain. Overall, the evidence may not support a “one-size-fits-all” approach. Future implementation could prioritize risk-stratified models targeting high-risk populations to potentially improve clinical and economic outcomes.

**Systematic review registration:**

The study protocol was registered with PROSPERO (CRD420251080763).

**Supplementary Information:**

The online version contains supplementary material available at https://doi.org/10.1186/s12889-026-28436-4.

## Introduction

General health checks (GHCs) are a widely implemented preventive strategy for adults in many high-income countries. The term “GHCs” encompasses a broad spectrum of practices, variously described as periodic health examinations or multiphasic screenings, and their implementation varies considerably in frequency, duration, and content [1, 2]. For the purpose of this review, GHCs are defined as broad operational preventive assessments for adults, aimed at synthesizing evidence across this heterogeneous landscape. The goal of such screening is to detect illness at an early stage and identify risk factors that can be modified, thereby triggering timely care to prevent later morbidity and mortality [3, 4]. However, implementing this at scale requires substantial resources and raises valid concerns about harms such as overdiagnosis, overtreatment, and the diversion of funds [5]. 

Several systematic reviews have evaluated the effects of GHCs, primarily focusing on Western high-income countries. An early review by Boulware et al. (2007) [6] suggested that periodic health evaluations improved the delivery of recommended preventive services and might reduce patient worry. Subsequent reviews have adopted a more cautious stance. The 2016 Cochrane review by Dyakova et al. [7], which focused on systematic cardiovascular risk assessment, found no statistically significant effects on major clinical endpoints, although modest improvements in cardiovascular risk factors were reported. Similarly, the 2019 Cochrane review by Krogsbøll et al. [4] concluded that GHCs were not associated with reductions in total or cardiovascular mortality. More recently, Liss et al. (2021) [2] reported no clear mortality benefit but found that GHCs were associated with increased detection of chronic conditions, improved risk factor control, and greater uptake of preventive services. These reviews have provided important evidence on the clinical effects of GHCs.

Despite these insights, important knowledge gaps remain. Prior syntheses were largely conducted in Western high-income settings and primarily focused on mortality and cardiovascular outcomes, with limited evaluation of psychological and economic outcomes. Moreover, several large-scale and long-term studies have been published since the most recent comprehensive review. Therefore, this systematic review was conducted to update and expand upon the work of Liss et al. [2]. By extending the search to 2025 and including multiple international and regional databases, this review incorporates findings from a broader range of studies, including evidence from developing countries. In addition to established clinical outcomes, we systematically evaluate psychological and economic outcomes to provide a more comprehensive assessment of the effects of GHCs.

## Methods

### Protocol and registration

The study protocol was registered with PROSPERO (CRD420251080763). This systematic review was reported in accordance with the Preferred Reporting Items for Systematic Reviews and Meta-Analyses (PRISMA) 2020 statement [8]. 

### Search strategy

We searched PubMed, Embase, the Cochrane Library, Web of Science, CINAHL via EBSCO, SinoMed, CNKI, and WanFang from database inception to July 1, 2025. All databases were searched on the same date to ensure consistency across the evidence base. The search strategy utilized a combination of controlled vocabulary (e.g., MeSH, Emtree) and free-text terms related to general health checks (e.g., “health check*,” “periodic health examination,” “multiphasic screening,” “physical examination”) and primary care settings. These were combined with study design filters for RCTs, NRCTs, observational studies with control groups, mixed-methods studies, and economic evaluations. To identify economic evaluations, the search strategy additionally incorporated specific keywords related to costs, cost-effectiveness, and economic modeling. Searches in Chinese databases utilized corresponding Chinese terms. The search was restricted to studies involving human adults and published in either English or Chinese. To ensure comprehensiveness, we also manually screened the reference lists of included articles and relevant systematic reviews [2, 4, 6, 7] to identify additional studies. The full electronic search strategies are detailed in the eAppendix in Additional file 1.

### Inclusion and exclusion criteria

Study eligibility criteria were established based on the PICOS (Population, Intervention, Comparator, Outcome, and Study design) framework (Table 1) [9]. 

**Table 1:** Inclusion and exclusion criteria based on the PICOS Framework

Criteria	Inclusion	Exclusion
Population	• Adults (≥ 18 years) from the general population in primary care or community settings	• Studies on special groups such as patients with dementia and autism
Intervention	• General health checks (GHCs): multi-component health assessments for individuals, including single, multiple, or periodic checks • May include brief interventions (e.g., health counseling) as an integral part of the GHC	• Screening programs limited to a single disease or single risk factor
Comparators	• No health check • Usual care • Alternative physical examination protocols	• Studies lacking an unexposed comparison group (e.g., single-arm studies or uncontrolled studies)
Outcome	Studies reporting ≥ 1 of the following: **Effectiveness Outcomes**: • Mortality: All-cause and cause-specific • Cardiovascular outcomes • Chronic disease diagnosis • Risk Factors Control • Healthcare Utilization • Health Behaviors • Patient-Reported Outcomes (PROs) • Psychological Outcomes **Economic Outcomes** • Total healthcare costs • High-cost services • Formal economic evaluations	• Studies not reporting any prespecified outcome of interest
Study design	• Randomized controlled trials (RCTs) • Nonrandomized controlled trials (NRCTs) • Observational studies with control groups (including prospective, retrospective, or historical cohort designs) • Economic evaluations • Mixed-methods studies	• Case series, case reports, editorials, reviews • Study protocols and ongoing trials • Articles for which full text was unavailable

### Literature selection and data extraction

Search results were imported into EndNote reference management software, and duplicates were removed. Three reviewers (Y.W., Q.Z., and X.Z.) independently screened titles and abstracts, with each record assessed by at least two reviewers. If an abstract indicated relevance to GHCs but did not report our prespecified outcomes, the study was retained for full-text review. Subsequently, full-text articles were assessed for eligibility based on the PICOS framework by two reviewers (Y.W. and Q.Z.). Disagreements at both the title/abstract screening and full-text assessment stages were resolved through discussion or consultation with a senior reviewer. Inter-reviewer agreement was assessed using Cohen’s kappa statistic at the full-text screening stage. To identify additional relevant studies, two reviewers (Y.W. and Q.Z.) independently screened the reference lists of included studies, while citation tracking was performed by X.Z.

Data extraction was performed independently by two reviewers (Y.W. and Q.Z.) using a standardized Microsoft Excel spreadsheet developed through group discussion and guided by the Cochrane Handbook for Systematic Reviews of Interventions [10]. In cases of missing data, corresponding authors were contacted. The following data were extracted: author (year), country, setting, study design, sample, intervention, comparison, follow-up duration, outcome of interest, and main findings.

### Risk of bias and quality assessment

Risk of bias was assessed using validated instruments tailored to specific study designs. RCTs were evaluated using the revised Cochrane Risk of Bias tool (RoB 2.0) [11] which assesses 5 domains to derive an overall judgment of low risk, some concerns, or high risk of bias. NRCTs were assessed using the ROBINS-I tool, covering 7 domains to categorize bias risk as low, moderate, serious, or critical [12, 13]. Cohort studies were appraised using the Newcastle-Ottawa Scale (NOS) [14], and cross-sectional studies were evaluated using the Joanna Briggs Institute (JBI) critical appraisal checklist [15]. Economic evaluation studies were assessed using Drummond’s checklist [16], which provides a framework for assessing the transparency, validity, and methodological quality of economic evaluations. Mixed-methods studies were evaluated using the Mixed Methods Appraisal Tool (MMAT) 2018 version [17], which contains separate criteria for qualitative, quantitative, and mixed-method components. Although the qualitative component was appraised as part of the MMAT assessment, only quantitative outcome data were extracted and included in the synthesis.

For these designs, methodological quality was categorized as high, moderate, or low based on prespecified scoring thresholds (eTable 1 in the Additional file 2). Two reviewers (Y.W. and Q.Z.) independently performed the assessments, with disagreements resolved by a third reviewer (X.Z.).

### Data synthesis

Given the substantial clinical and methodological heterogeneity across studies, including differences in populations, intervention components, study designs, follow-up duration, and outcome definitions, we explored whether meta-analysis might be feasible for any subset of studies; however, pooling was ultimately considered inappropriate.

Therefore, a structured narrative synthesis was undertaken in accordance with the Synthesis Without Meta-analysis (SWiM) reporting guideline [18] and guidance from Chap. 12 of the Cochrane Handbook for Systematic Reviews of Interventions [19]. Studies were grouped according to predefined outcome domains. Within each outcome domain, findings were interpreted according to study design, with particular attention to differences between randomized and observational studies. Differences in follow-up duration and population characteristics, such as high-risk populations and older adults, were considered when interpreting the results. In addition, the direction, magnitude, and statistical significance of reported effects were compared descriptively across studies, with particular attention to discrepancies between randomized and nonrandomized evidence.

Due to the absence of statistical pooling, formal subgroup and sensitivity analyses were not conducted. Publication bias was not assessed using funnel plots because such methods require meta-analytic estimates. However, comprehensive searches across multiple international and regional databases, including Chinese databases, and screening of reference lists were performed to reduce the risk of reporting bias.

### Certainty of evidence

We assessed the certainty of evidence for each outcome using the Grading of Recommendations Assessment, Development and Evaluation (GRADE) approach. [20, 21] For outcomes including both randomized and nonrandomized studies, we followed GRADE guidance to derive an overall certainty rating across study designs. [22, 23] In the absence of meta-analysis, we applied the approach proposed by Murad et al. [24] to evaluate risk of bias, inconsistency, indirectness, imprecision, and publication bias. All certainty ratings and reasons for downgrading or upgrading are presented in the footnotes of the GRADE evidence profile.

### Deviations from the registered protocol

This review was conducted in accordance with the registered protocol. However, two deviations from the original protocol are reported.

First, although quantitative meta-analysis was initially planned, statistical pooling was not performed due to substantial heterogeneity in study design, intervention characteristics, and outcome measures. A single pooled estimate was therefore not appropriate.

Second, the appraisal tool for economic evaluations was changed from the CHEERS checklist to the Drummond checklist to better assess methodological quality rather than reporting quality.

## Results

### Study selection

**Fig. 1:**
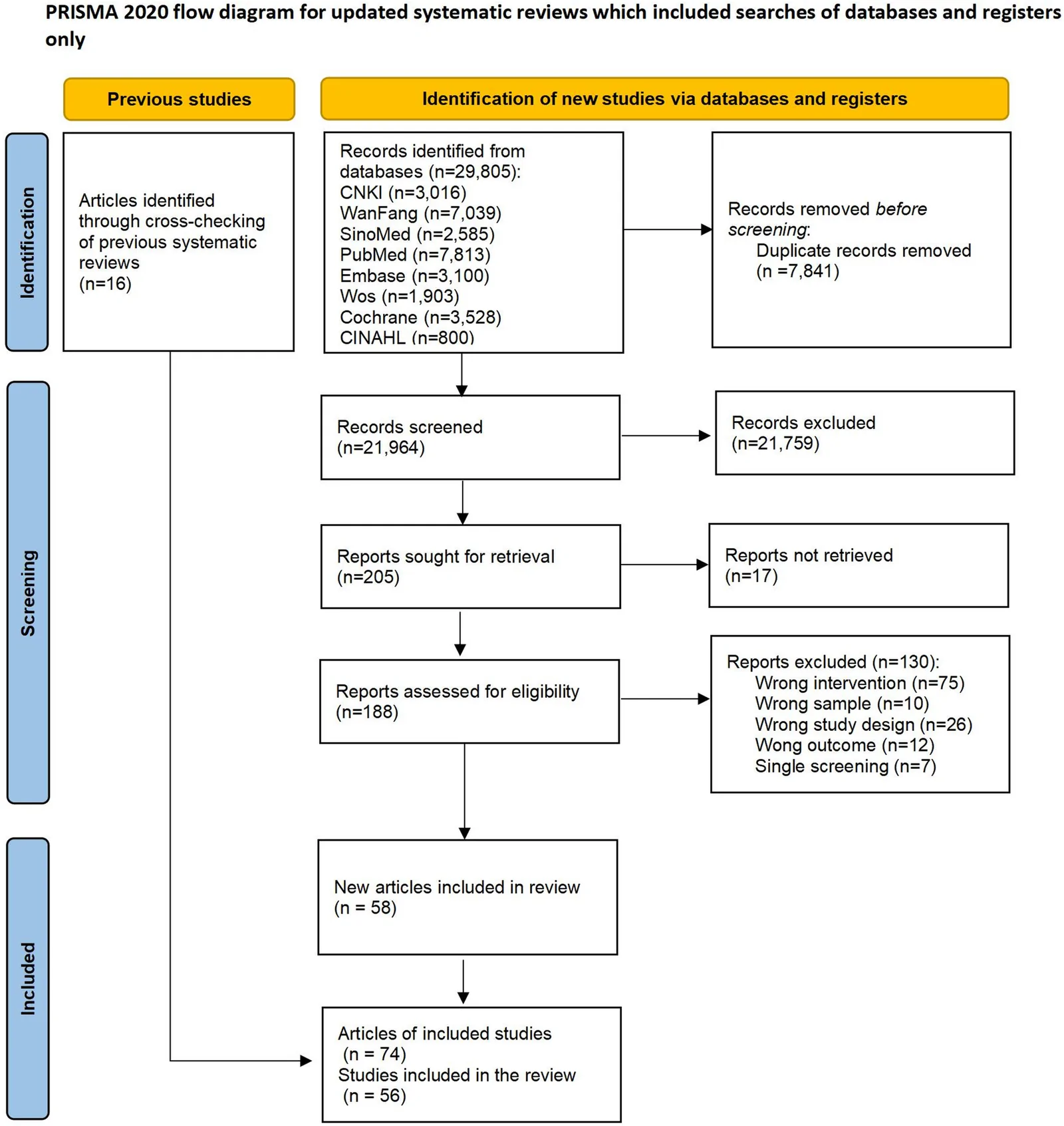
Flowchart of study selection

As shown in Fig. 1, the systematic search across 8 databases identified 29,805 initial records. After removing 7,841 duplicates, 21,964 records remained for screening. Based on titles and abstracts, 21,759 records were excluded. Full texts were sought for the remaining 205 records; 17 could not be retrieved, and 130 were excluded after full-text review based on the eligibility criteria. Cohen’s kappa for inter-reviewer agreement at the full-text screening stage was 0.83, indicating substantial agreement. This process yielded 58 articles. To ensure comprehensiveness, we subsequently cross-checked the reference lists of four previous systematic reviews on this topic, including that by Liss et al. [2, 4, 6, 7]. Through this manual screening, we identified 16 additional articles that met the eligibility criteria but were not captured by the electronic search strategy. Many of these were older seminal trials published before the term “general health checks” became widely used or were indexed under alternative terminology not consistently captured by electronic search strategies. In total, 74 articles representing 56 unique studies were included in this updated systematic review. Of the 56 studies, 32 had been identified in previous reviews [2, 4, 6, 7], whereas 24 were newly identified through our updated search.

### Study characteristics

The review included 22 RCTs [25–63], 19 cohort studies [64–83], 6 cross-sectional studies [84–89], 5 NRCTs [90–94], 3 economic evaluation studies [95–97], and 1 mixed-methods study [98]. Each study’s characteristics are summarized in eTable 2 in the Additional file 3. The 56 included studies comprised more than 17,145,442 participants, with publication dates spanning 52 years (1973–2025). Follow-up duration ranged from 2 months to 30 years, with 1 modeling study employing a lifetime simulation horizon (up to 50 annual cycles).

**Fig. 2:**
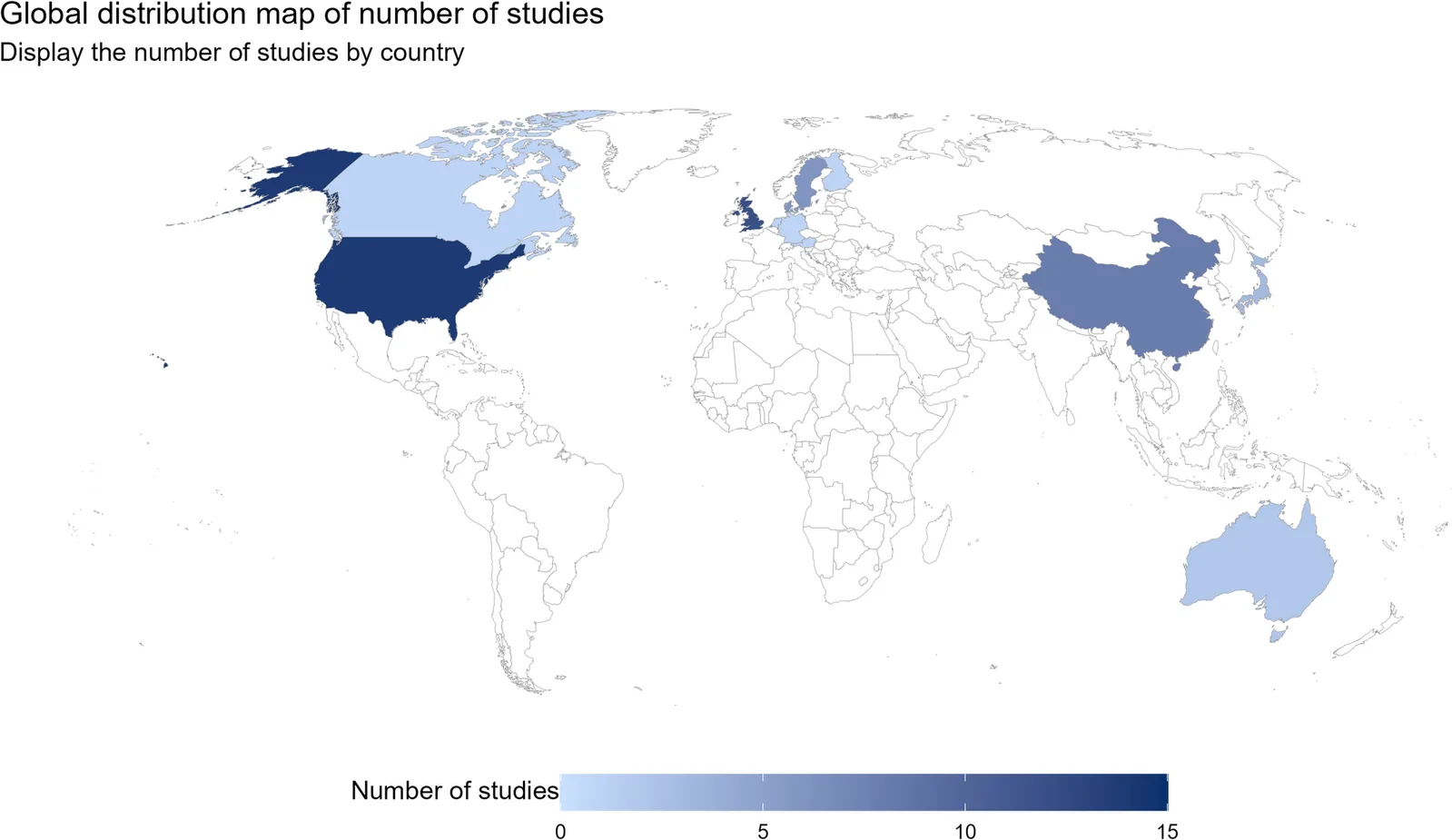
Global distribution and number of included studies by country

The included studies were conducted in 12 countries across North America, Europe, and Asia. The majority originated from high-income countries. The United States contributed the most studies (14 [25.0%]), followed by the United Kingdom (12 [21.4%]), China (8 [14.3%]), Denmark (6 [10.7%]), and Sweden (6 [10.7%]) (Fig. 2). Most studies were set in primary care practices, with others based in community or workplace settings. Participant ages ranged from 18 to 100 years, and 13 studies (23.2%) were restricted to older adults (aged ≥ 65 years). Compared with earlier reviews dominated by North American data [2], the current sample included a broader range of European and Asian studies, although evidence from low- and middle-income countries remained scarce.

### Risk of bias and quality assessment

**Fig. 3:**
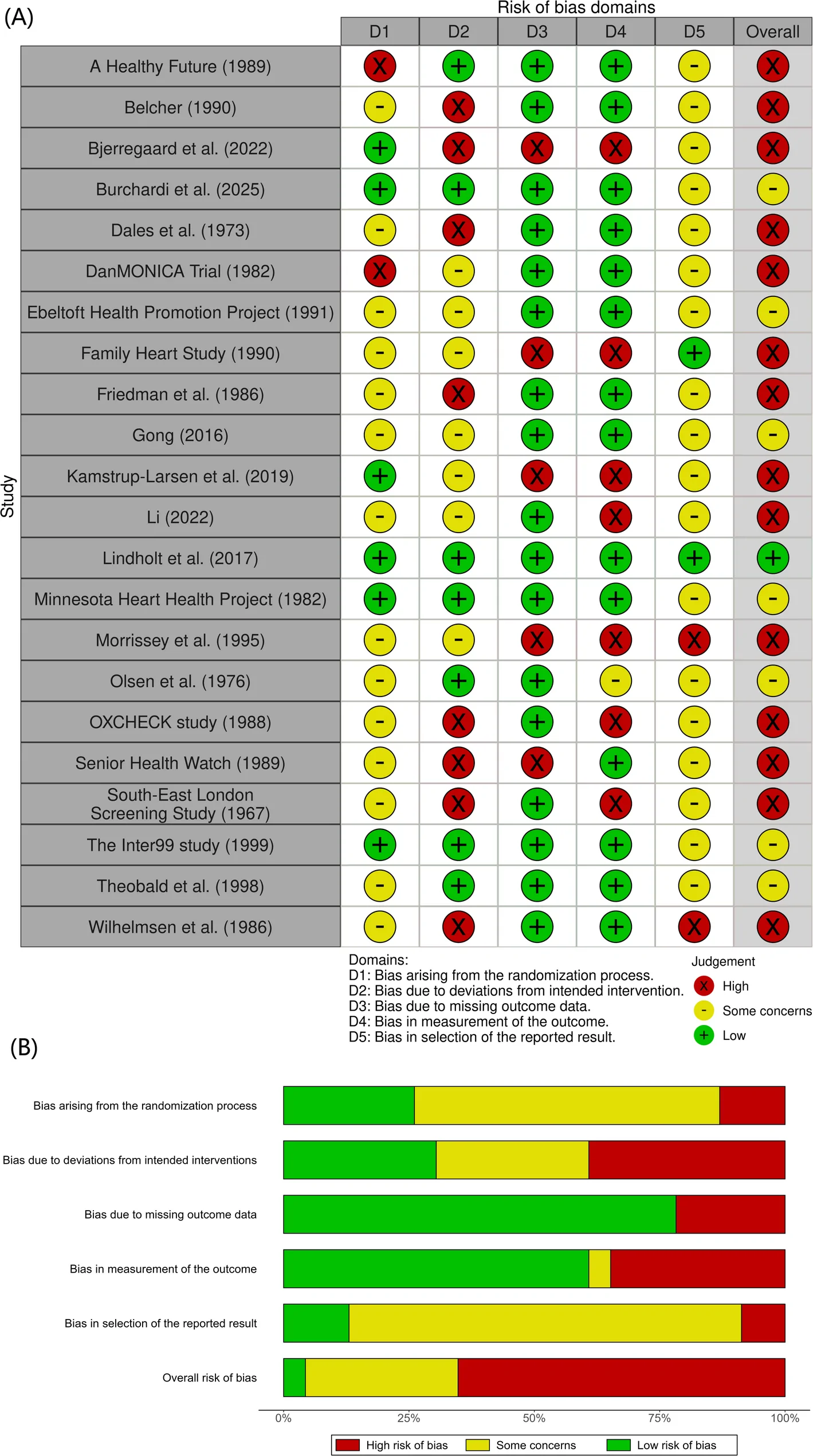
Risk of bias in randomized controlled trials of general health checks. (**A**) Study-level overall risk-of-bias judgments for each randomized controlled trial. (**B**) Domain-specific distribution of risk-of-bias judgments across all randomized controlled trials

Among the 22 RCTs, 1 (4.5%) was rated as having a low risk of bias, 7 (31.8%) as having some concerns, and 14 (63.6%) as having a high risk of bias (Fig. 3). Of the 5 NRCTs, 2 (40.0%) had a moderate risk of bias, and 3 (60.0%) had a serious risk. Regarding methodological quality, 18 of 19 cohort studies (94.7%) were rated as high quality, and 1 (5.6%) was rated as moderate quality. Of the 6 cross-sectional studies, 4 (66.7%) were rated as high quality and 2 (33.3%) as moderate quality. Two of the three economic evaluation studies were rated as high quality, whereas one was rated as low quality. The single mixed-methods study was rated as moderate quality. Detailed assessment results for each study design are presented in eTable 3–8 in the Additional file 3.

### Effectiveness of general health checks

The findings of GHCs across various outcome domains are summarized in Table 2. The following sections provide a detailed evaluation of each domain.

**Table 2:** Summary of findings across outcome domains

Outcome domain	No. of studies	Summary of findings
Mortality	22 studies (13 RCTs, 8 observational studies, 1 economic evaluation study)	No significant reduction in all-cause mortality in the general population; however, modest benefits were observed in specific high-risk or older subgroups.
Cardiovascular events	9 studies (4 RCTs, 2 NRCTs, 2 observational studies, 1 economic evaluation study)	No significant reduction in cardiovascular events in RCTs, contrasting with potential protective associations in observational studies.
Chronic disease diagnosis	13 studies (3 RCTs, 2 NRCTs, 8 observational studies)	Consistently higher detection of some chronic diseases and depression across all study designs.
Risk Factor Control	16 studies (8 RCTs, 2 NRCTs, 6 observational studies)	The majority of trials and observational studies reported modest improvements in blood pressure, lipid profiles, and cardiovascular risk scores; although some trials found null effects.
Healthcare Utilization	23 studies (12 RCTs, 1 NRCT, 10 observational studies	Increased uptake of preventive services and medications, however, generally did not demonstrate a reduction in hospital utilization.
Health Behaviors	13 studies (8 RCTs, 2 NRCTs, 2 observational studies, 1 mixed-methods study)	Improved diet and physical activity, but RCTs found no effect on smoking or alcohol, contrasting with positive associations in observational studies.
Patient-Reported Outcomes (PROs)	11 studies (9 RCTs, 1 NRCT, 1 observational study)	Most studies reported modest improvements in quality of life or patient satisfaction, although two studies observed no significant changes.
Psychological Outcomes	10 studies (7 RCTs, 3 observational studies)	Mental health effects were positive or neutral, but one cohort study reported an increased risk of suicide in older adults.
Economic Outcomes	14 studies (6 RCTs, 5 observational studies, 3 economic evaluation studies)	Associated with reductions in short-term healthcare costs; however, formal economic evaluations indicated that long-term cost-effectiveness varied drastically by country.

#### Mortality

GHCs were not consistently associated with changes in mortality. A total of 22 studies reported at least one mortality outcome, including 13 RCTs [27, 34, 36, 38, 39, 44, 45, 47, 51, 52, 54, 55, 60, 62, 63], 8 observational studies [68, 71, 73, 74, 76, 77, 82, 83], and 1 economic evaluation [95]. Overall, 10 of the 13 RCTs found no significant reduction in all-cause mortality compared with usual care among the general population [27, 34, 36, 38, 47, 51, 52, 54, 55, 60]. For example, in a large Swedish trial (*n* = 32,186), a one-time combined medical, psychological, and social assessment was not associated with a significant difference in 22-year all-cause mortality (RR, 1.03; 95% CI, 0.94–1.14), cardiovascular mortality, cancer mortality, or accidental death after adjustment [38]. Similarly, the Ebeltoft Health Promotion Project (*n* = 3,464) found no difference in 25-year all-cause mortality between invitees and non-invitees (HR, 0.93; 95% CI, 0.75–1.16) [34]. Notably, 2 of these trials even suggested potential harm. In the A Healthy Future trial (*n* = 2,558), mortality was higher in the group offered a 2-year preventive services package (9.8% vs. 8.2%; *P* = .06), particularly among participants aged 75 years or older (18.6% vs. 13.5%; *P* = .05) [51]. Similarly, in the Inter99 study (*n* = 59,616), women in intervention areas with high participation had a 32% higher risk of death over 10 years compared with controls (HR, 1.32; 95% CI, 1.03–1.69) [62], driven by lifestyle-related and cancer-related mortality [63]. 

However, modest mortality benefits were observed in 4 RCTs [39, 44, 45, 52] targeting high-risk or older populations and in 6 high-quality cohort studies [68, 74, 76, 77, 82, 83]. For instance, one early trial (*n* = 30,022) reported that a high-risk subgroup of men (baseline SBP ≥ 175 mm Hg) had 40% lower total mortality and 30–37% lower cardiovascular mortality compared with historical controls (*P* < .001) [52]. Regarding older adults, the remaining three trials also observed small but statistically significant reductions in mortality [39, 44, 45]. For example, in the Senior Health Watch trial (*n* = 4,195), older participants receiving preventive and counseling visits had lower mortality than those in usual care at 2 years (8% vs. 11%; *P* = .003) and 6 years (19% vs. 22%; *P* = .02) [39]. Consistent with these trial findings, a large Chinese cohort study involving 625,279 older adults also found that regular health checks were associated with substantially lower cardiovascular mortality (HR, 0.44; 95% CI, 0.43–0.45) and all-cause mortality (HR, 0.44; 95% CI, 0.44–0.45), with stronger associations among those with diabetes, dyslipidemia, or hypertension [74]. 

#### Cardiovascular outcomes

A total of 9 studies evaluated cardiovascular outcomes [34, 52, 54, 60, 72, 76, 91, 93, 97]. Strikingly, all 4 RCTs [34, 52, 54, 60] and 2 NRCTs [91, 93] consistently failed to show a significant reduction in the incidence of cardiovascular events among the general population. In the DanMONICA trial (*n* = 17,845), participants in the intervention group were invited to up to 3 health checks, yet the 30-year incidence of ischemic heart disease did not differ significantly from that in the usual care group (HR, 0.99; 95% CI, 0.92–1.07); notably, the incidence of stroke was significantly higher in the health check group (HR, 1.14; 95% CI, 1.04–1.25) [54]. However, benefits were identified in specific high-risk subgroups. An early influential trial showed that although physical examination could not reduce the cardiovascular incidence rate of ordinary men, in the high-risk subgroup with baseline systolic blood pressure ≥ 175 mm Hg, the 10-year stroke incidence rate decreased by 30% [52]. 

In contrast to the evidence from controlled trials, the observational studies [72, 76] and an economic evaluation [97] suggested a potential protective associations. In a Japanese study of annual physical examinations among adults aged 65 years and older, compared to those who participated in the examination, non-participation was specifically associated with increased stroke-related certification in younger older men (65–74 years), suggesting a protective effect of participation [72]. Similarly, a high-quality British cohort found that, over the longer term (≥ 2 years), health checks were associated with significant reductions in the incidence of myocardial infarction (HR, 0.85; *P* < .05) and atrial fibrillation (HR, 0.91; *P* < .05) [76]. 

#### Chronic disease diagnosis

A total of 13 studies reported that general health checks were associated with higher detection of chronic conditions [27, 36, 43, 64, 67, 72, 75, 76, 78, 79, 86, 92, 93], including 3 RCTs [27, 36, 43], 2 NRCTs [92, 93] and 8 observational studies [64, 67, 72, 75, 76, 78, 79, 86]. For somatic diseases, a UK cohort of 138,788 participants compared with nonparticipants, NHS health check participants had a 3.3% point increase in hypertension diagnosis, a 1.4% point increase in type 2 diabetes diagnosis, and a 0.2% point increase in chronic kidney disease diagnosis [64]. Notably, this improved detection extends beyond somatic conditions to mental health. Three studies have found that physical examinations are associated with a higher rate of newly diagnosed depression [43, 67, 75]. Interestingly, while a Danish trial found no significant difference in the detection of hypertension, hypercholesterolemia, diabetes, or COPD, it reported that the diagnosis rate of depression in the intervention group was more than twice that of the control group (OR, 2.90; 95% CI, 1.34–6.29) [43]. A study in Taiwan reported that the rate of newly treated depression in the screening group was 63% higher (HR, 1.63; 95% CI, 1.62–1.64) [67]. Similarly, a US study found that completion of physical examinations during the COVID-19 pandemic was significantly associated with a higher diagnosis rate of depression compared with controls [75]. 

#### Risk factor control

Among 16 studies evaluating risk factor control [25, 29, 35, 43, 46, 49, 52, 55, 64, 66, 69, 80, 89, 90, 93, 94], 13 reported improvements in blood pressure, lipid profiles, or composite cardiovascular risk scores associated with general health checks [25, 29, 35, 46, 49, 52, 64, 69, 80, 89, 90, 93, 94]. These included 6 RCTs [25, 29, 35, 46, 49, 52], 2 NRCTs [93, 94], and 5 observational studies [64, 69, 80, 89, 90]. In the Family Heart Study (*n* = 12,472), participation in the health check, compared with nonparticipation, was associated with decreased Dundee coronary risk score. Systolic blood pressure decreased by 6.2 mm Hg in women and 7.3 mm Hg in men, and diastolic blood pressure decreased by 3.0 mm Hg in women and 3.5 mm Hg in men [35]. Similarly, a hospital-based vascular screening program in the Netherlands (*n* = 1170) demonstrated that systolic blood pressure decreased by 2.5 mm Hg and LDL cholesterol levels decreased by 0.3 mmol/L among participants who underwent screening compared with those receiving usual care [90]. Conversely, 3 studies reported no significant differences in risk factor control between intervention and control groups [43, 55, 66]. 

#### Healthcare utilization

A total of 6 RCTs [26–28, 42, 47, 51] and 4 observational studies [66, 75, 87, 88] reported higher utilization of clinical preventive services. In a German RCT (*n* = 1,401), participants in the check-up 45 + group applied for rehabilitation and prevention services significantly more often than those receiving usual care (12.0% vs. 2.9%; *P* < .001), and higher proportions had services approved and completed (8.4% vs. 2.4%; 7.2% vs. 2.3%; *P* < .001 for both) [26]. Similarly, in a Canadian study of women aged 20 years or older (*n* = 2,332), those attending periodic health examinations were more likely than those without regular checks to receive Pap smears (OR, 6.7; 95% CI, 4.6–9.8), mammograms (OR, 3.7; 95% CI, 2.3–5.9), bone densitometry (OR, 3.7; 95% CI, 1.3–10.5), and cholesterol testing (OR, 3.0; 95% CI, 2.0-4.5), with testing rates increasing in correlation with the frequency of annual physician visits [87]. 

Regarding medication use, 3 RCTs [27, 45, 52], 1 NRCT [93], and 5 observational studies [64, 69, 78–80] reported increased initiation of preventive pharmacotherapy associated with GHCs. In the Viborg Vascular (VIVA) trial (*n* = 50,156), participants in the screening group were significantly more likely to initiate antithrombotic (HR, 2.30), lipid-lowering (HR, 2.10), and antihypertensive therapy (HR, 1.57) compared with controls [45]. Similarly, in a large UK cohort (*n* = 3,492,816), NHS Health Check attendees were more likely than nonattendees to be prescribed statins (HR, 2.98; 95% CI, 2.84–3.13) and antihypertensive medications (HR, 1.65; 95% CI, 1.59–1.72) [79]. 

By contrast, GHCs were not consistently associated with reduced hospital utilization; a total of 6 RCTs [27, 31, 36, 45, 48, 55] and 2 observational studies [66, 73] evaluated hospital utilization. Overall, the RCTs generally did not demonstrate a reduction in utilization, with findings ranging from null effects to increased burdens. Three RCTs [31, 36, 55] and one observational study [66] found no significant difference in total hospital days or admission rates.

Some trials showed more complex patterns. One trial found that hospital usage was slightly lower among men but slightly higher among women, yielding no overall benefit [27]. Similarly, the VIVA trial showed that fewer inpatient days for peripheral arterial disease and hypertension were offset by increases for chronic obstructive pulmonary disease and stroke, resulting in no net reduction in hospital burden [45]. Notably, Olsen et al. [48] even observed significantly more hospital nights among screened participants at 1 year, a finding potentially linked to poor follow-up on detected abnormalities.

However, one exception was a Finnish cohort study [73], which observed a substantial 67% reduction in ED visits (IRR, 0.33; *P* < .001) in the intervention group over three years. The authors attributed this finding to preventive management and early detection of specific conditions, although they also noted possible selection bias and baseline differences between participants and non-participants.

#### Health behaviors

A total of 13 studies evaluated health behaviors [35, 40, 43, 46, 49, 51, 52, 55, 69, 80, 93, 94, 98], including 8 RCTs [35, 40, 43, 46, 49, 51, 52, 55], 2 NRCTs [93, 94], 2 observational studies [69, 80], and 1 mixed-methods study [98]. Results varied significantly by the type of behavior targeted and study design. Studies evaluating diet and physical activity commonly observed positive outcomes. For example, in the Minnesota Heart Health Project (*n* = 847), participants receiving a screening and education program had significantly higher physical activity levels (3,081 vs. 2,401 kcal/week; *P* < .01) and were more likely to select low-fat and low-sodium foods compared with controls [46]. 

However, for the two addictive behaviors of smoking and alcohol consumption, findings diverged notably according to study design. Strikingly, 7 of 8 RCTs evaluating these behaviors consistently indicated that GHCs did not reduce smoking or alcohol consumption [35, 40, 43, 46, 49, 51, 55]. For example, in a Danish RCT among adults aged 45 to 64 years (*n* = 1,104), no significant differences were observed at 12 months between intervention and control groups in daily smoking (OR, 0.99; 95% CI, 0.76–1.30) or binge drinking (OR, 0.82; 95% CI, 0.59–1.14) [43]. 

In contrast, all 5 nonrandomized studies [69, 80, 93, 94, 98] reported positive associations. In a high-quality UK cohort (*n* = 450,801), health check participants had a lower smoking prevalence after 6 years compared with nonparticipants (OR, 0.90; 95% CI, 0.87–0.94) [80]. In a study of older adults in China, the management group showed significantly greater reductions in alcohol consumption compared with controls (10.0% vs. 28.0%; *P* < .05) [94]. 

#### Patient-Reported Outcomes (PROs)

A total of 11 studies [28, 42, 44, 45, 47, 48, 51, 55, 59, 84, 94] evaluated patient-reported outcomes, including quality of life, self-rated health, and patient satisfaction. Among these, 9 studies reported small improvements in PROs associated with GHCs, including 7 RCTs [28, 42, 44, 45, 47, 51, 59], 1 NRCT [94], and 1 observational study [84]. For example, an RCT conducted in the United States (*n* = 1914) reported modest improvements in multiple quality-of-life measures in the intervention group compared with the control group over a 2-year follow-up period [47]. Conversely, 2 trials—the Multiphasic Health Checkup Evaluation Study [48] and the South-East London Screening Study [55] — found no significant differences in self-reported health status or quality of life between intervention and control groups.

#### Psychological outcomes

A total of 10 studies evaluated mental health outcomes [25, 33, 43, 45, 51, 56, 58, 59, 67, 75, 84], including 7 RCTs [25, 33, 43, 45, 51, 56, 58, 59] and 3 observational studies [67, 75, 84]. Overall, GHCs demonstrated predominantly positive or neutral effects on depression, anxiety, or psychological distress. In the Inter99 study, health checks did not increase distress; instead, they were associated with temporary alleviation of anxiety, depression, and somatization, with some improvements persisting for up to 10 months [58], as well as longer-term improvements in physical and mental health over a 5-year follow-up period. (*P* < .001) [59]. Additionally, several studies reported that health checks were associated with higher detection rates of depression [43, 67, 75]. However, a high-quality cohort study from Taiwan (*n* = 9,584,456) revealed a complex association: screening was linked to a reduced risk of psychiatric hospitalization (HR, 0.93; 95% CI, 0.91–0.95) but, conversely, a higher risk of suicide among adults aged 65 years or older [67]. Finally, potential psychological benefits appeared transient; the Ebeltoft Health Promotion Project found no measurable psychological effects after 25 years of follow-up [33]. 

### Economic outcomes

#### Total healthcare costs

A total of 14 studies evaluated economic outcomes [28, 33, 40, 47, 51, 55, 66, 71–73, 81, 95–97], including 6 RCTs [28, 33, 40, 47, 51, 55], 5 observational studies [66, 71–73, 81], and 3 formal economic evaluation studies [95–97]. Overall, GHCs were associated with reductions in total healthcare costs. For example, in the Senior Health Watch trial, the intervention group showed a 2.5% reduction in total medical insurance costs in the second year compared with the control group [40]. Another study similarly reported that participants receiving annual wellness visits experienced a 5.7% decrease in total healthcare costs within 11 months compared with nonparticipants [66]. Additionally, 2 long-term US reports found per capita net savings of US$190 over 3 years [47] and US$822 over 7 years [95] among individuals attending health checks.

#### High-cost services

GHCs were associated with reduced utilization of high-cost services, particularly emergency department (ED) visits and hospitalizations, which are also key drivers of cost savings. In a Finnish study of adults aged 75 years or older, the emergency visit rate in the screening group decreased by 67% over 3 years (incidence rate ratio [IRR], 0.33; 95% CI, 0.17–0.63), and ED costs decreased from €53 to €8 per person-year (mean ratio, 0.15; 95% CI, 0.10–0.71). In contrast, the control group experienced a 19% increase in emergency visits (IRR, 1.19; 95% CI, 1.08–1.31) and an increase in ED costs from €148 to €183 (mean ratio, 1.24; 95% CI, 1.08–1.40) [73]. 

#### Formal economic evaluations

Formal economic evaluations provided divergent findings regarding cost-effectiveness. A UK analysis estimated that health checks yielded an incremental gain of 0.05 quality-adjusted life-years (QALYs) while reducing disease-related costs by £170. The calculated incremental cost-effectiveness ratio (ICER) was £900 per QALY gained, suggesting high cost-effectiveness [96]. In contrast, a study from Australia presented less favorable results. While health checks increased lifetime QALYs (0.008 for males; 0.003 for females), the estimated ICERs were AU$42,355 per QALY gained for males and AU$133,504 for females. Probabilistic sensitivity analysis demonstrated that, at a willingness-to-pay threshold of AU$28,000 per QALY, the probability of cost-effectiveness was only 17.5% for males and 0% for females, suggesting that GHCs were unlikely to be cost-effective in this specific setting [97]. Formal economic evaluations produced heterogeneous results across settings.

### GRADE evidence profile

The GRADE evidence profile for all evaluated outcome domains, including detailed assessments of the domains considered for downgrading, is presented in Table 3. Certainty of evidence ranged from moderate to very low across outcome domains, with most outcomes rated as low certainty.

**Table 3:** GRADE Evidence Profile: GHCs compared with usual care or no screening in adults

Outcome	Risk of bias	Inconsistency	Indirectness	Imprecision	Other considerations	Overall certainty
Mortality	Serious^a^	Not Serious	Not Serious	Not Serious	None	⨁⨁⨁◯ Moderate
Cardiovascular events	Serious^a^	Not Serious	Not Serious	Serious^b^	None	⨁⨁◯◯ Low
Chronic disease diagnosis	Serious^a^	Not Serious	Not Serious	Not Serious	None	⨁⨁⨁◯ Moderate
Risk Factor Control	Serious^a^	Not Serious	Not Serious	Serious^b^	None	⨁⨁◯◯ Low
Healthcare Utilization	Serious^a^	Not Serious	Not Serious	Not Serious	None	⨁⨁⨁◯ Moderate
Health Behaviors	Serious^a^	Seriousc^c^	Not Serious	Not Serious	None	⨁⨁◯◯ Low
Patient-Reported Outcomes	Serious^a^	Not Serious	Not Serious	Serious^b^	None	⨁⨁◯◯ Low
Psychological Outcomes	Serious^a^	Serious^d^	Not Serious	Not Serious	None	⨁⨁◯◯ Low
Economic Outcomes	Serious^a^	Serious^e^	Serious^f^	Not Serious	None	⨁◯◯◯ Very Low

GRADE Working Group grades of evidence:

XHigh certainty: we are very confident that the true effect lies close to that of the estimate of the effect

Moderate certainty: we are moderately confident in the effect estimate: the true effect is likely to be close to the estimate of the effect, but there is a possibility that it is substantially different

Low certainty: our confidence in the effect estimate is limited: the true effect may be substantially different from the estimate of the effect

Very low certainty: we have very little confidence in the effect estimate: the true effect is likely to be substantially different from the estimate of effect

^a^ Risk of Bias: Downgraded one level. The majority of included RCTs were rated as having a high risk of bias or some concerns

^b^ Imprecision: Downgraded one level. While the overall direction favored intervention or null, findings included a mix of significant and non-significant results without completely opposing directional conflict

^c^ Inconsistency: Downgraded one level. Results demonstrated conflicting directional findings across study designs, with randomized and nonrandomized evidence pointing toward opposing conclusions

^d^ Inconsistency: Downgraded one level. While RCTs predominantly showed neutral or positive effects, a conflicting signal of potential harm was observed in a specific observational cohort

^e^ Inconsistency: Downgraded one level. Formal economic evaluations produced highly heterogeneous and divergent results regarding cost-effectiveness, ranging from highly cost-effective in one setting to not cost-effective in another

^f^ Indirectness: Downgraded one level. Cost-effectiveness metrics varied drastically as they are highly dependent on country-specific healthcare systems, currencies, and willingness-to-pay thresholds, making the findings highly indirect across different global settings

## Discussion

In this comprehensive systematic review, we provide an updated evaluation of GHCs across multiple outcome domains. We found no consistent evidence that routine GHCs reduce all-cause mortality, major cardiovascular events, or hospitalizations, especially in large-scale, long-term RCTs. Conversely, GHCs appeared to be consistently associated with increased detection of chronic diseases, modestly improved risk factor control, and greater uptake of preventive services and pharmacotherapy. While the impact on health behaviors was mixed, patient-reported outcomes suggested modest improvements. Importantly, this synthesis covers two frequently neglected areas: economic outcomes and mental health. We found that while the psychological outcomes were generally positive or neutral, potential risks may exist, especially for older adults. Furthermore, we identified evidence linking GHCs to cost savings and potential cost-effectiveness in specific settings, which may challenge long-held assumptions about their purely additive economic burden. Importantly, both the direction and magnitude of effects differed across study designs, baseline risk profiles, and intervention components, suggesting that how and to whom health checks are delivered may be more important than simply whether they are offered. Although the direction of effects was relatively consistent for some intermediate outcomes, the overall certainty of evidence ranged from moderate to very low, underscoring the need for cautious interpretation.

Although modest improvements in cardiovascular risk factors, such as blood pressure and lipid levels, were observed, significant reductions in all-cause mortality and cardiovascular events appeared to be absent in the general population. This finding aligns with previous major systematic reviews. However, our analysis offers critical nuance. The mortality benefits observed exclusively in high-risk or older populations [39, 44, 45, 52] likely represent scenarios where baseline risk is sufficient for modest risk factor improvements to translate into a statistically detectable survival advantage. Importantly, these subgroup findings were generally based on pre-specified analyses or trials designed a priori to enroll higher-risk or older populations, rather than solely on post hoc exploratory observations. This pattern may also reflect the time lag between improvements in risk factors and measurable effects on hard clinical outcomes. In populations at relatively low baseline risk, the magnitude of risk reduction may be insufficient to produce observable differences in survival during the follow-up periods typical of randomized trials.

This interpretation is also consistent with our findings on cardiovascular events: while RCTs in general populations showed no benefit [34, 45, 52, 54, 60], studies of older adults or high-risk subgroups suggested potential long-term reductions in myocardial infarction and stroke [52, 72]. Collectively, these findings suggest that GHCs are unlikely to yield survival benefits in unselected adult populations but may be clinically meaningful when targeted at older adults or individuals with elevated cardiovascular risk. From a public health perspective, these findings may support more risk-stratified approaches to health checks rather than uniform population-wide implementation. Preventive resources may be more effectively directed toward older adults and individuals at higher baseline cardiovascular risk, who appeared more likely to benefit from screening interventions in the included studies.

Our findings suggest that the potential value of GHCs may lie less in reducing mortality directly and more in facilitating entry into preventive care. We observed that these checks excel at case-finding, driving up diagnosis rates for a wide range of chronic issues, from diabetes and hypertension to depression [27, 36, 43]. Once these conditions are spotted, clinical action typically follows: screened participants were consistently more likely to start necessary preventive pharmacotherapies [27, 45, 52] and receive preventive services [26–28, 42, 47, 51]. These findings suggest that the effectiveness of GHCs may depend not only on the health check itself but also on the availability of appropriate follow-up and long-term management within primary care systems. Accordingly, GHCs may be more effective when implemented as integrated entry points into broader chronic disease prevention and management systems rather than as standalone screening events.

Findings related to health behaviors were mixed. While health checks appeared to encourage better diet and physical activity, RCTs consistently showed they had little impact on smoking or alcohol use. These findings suggest that brief counseling during health checks may support modest lifestyle changes but may be insufficient to substantially change addictive behaviors, which likely require more intensive, specialized help. On the other hand, patients generally reported better quality of life and satisfaction, suggesting they personally value the reassurance these assessments provide. However, potential psychological harms should also be considered. While most findings on anxiety and depression were neutral or positive, the signal of increased suicide risk in certain older populations points to a potential double-edged effect [67]. These findings suggest that psychological assessment and follow-up may warrant greater attention in preventive health programs, particularly for older adults; structured mental health support and suicide risk monitoring may be important components of follow-up care.

An important aspect of this updated review is the inclusion of economic outcomes, which were less extensively evaluated in earlier reviews. We found evidence suggesting that GHCs may be associated with short-term reductions in overall healthcare costs, potentially related to reduced use of emergency and hospital services [66–73]. However, findings related to long-term cost-effectiveness were inconsistent across settings. Economic models from the UK suggested that GHCs may be cost-effective, showing ratios well below standard willingness-to-pay thresholds [96]. In contrast, Australian studies suggested that routine checks may be less cost-effective in the general population, especially among women [97]. This divergence reflects differences in baseline healthcare costs, population risk profiles, and the design and frequency of the health checks themselves, highlighting that cost-effectiveness depends heavily on the specific context and policies. From a policy perspective, these findings suggest that GHCs should not necessarily be viewed solely as an additional healthcare cost. Instead of seeing GHCs purely as a drain on resources, they may be more appropriately considered as a strategic investment that can improve resource use, particularly when targeted toward higher-risk populations and integrated with primary care and chronic disease management. At the same time, equity considerations should be kept in mind to ensure that the benefits of health checks are accessible across different population subgroups.

We also observed notable differences between findings from randomized trials and observational studies. RCTs generally reported little or no effect on major cardiovascular events or mortality [27, 34, 36, 38, 45, 47, 51, 52, 54, 55, 60]. Yet, observational studies more frequently suggested protective associations among attendees, particularly among older adults or those with cardiometabolic risks [68, 72, 74, 76, 77, 82, 83]. A similar pattern was seen for health behaviors: RCTs demonstrated little to no impact on smoking or hazardous alcohol use [35, 40, 43, 46, 49, 51, 52, 55], whereas observational studies reported lower smoking prevalence and more favorable alcohol-related behaviors among attendees [69, 80]. These discrepancies likely reflect residual confounding, the “healthy user effect,” and socioeconomic factors influencing participation in health checks. As a result, observational studies may overstate the causal benefits of GHCs, meaning that RCT evidence should remain the primary basis for evaluating the effectiveness of GHCs in the general population.

Several studies also reported potentially adverse or ambivalent findings that warrant careful consideration. In the A Healthy Future trial [51], mortality was higher among intervention participants aged 75 years or older compared with controls; however, a subsequent analysis attributed this difference largely to greater use of advance directives and less life-prolonging treatment at the end of life [50], suggesting that the observed difference may reflect end-of-life care preferences rather than direct harm from the intervention. In the Inter99 trial [62], women in intervention areas with high participation had higher all-cause mortality, and investigators hypothesized that widespread use of nutritional supplements might have contributed to smoking-related cancers, although this remains uncertain. In the DanMONICA trial [54], invitation to repeated health checks was associated with a higher long-term incidence of stroke, which the authors linked to potential mechanisms including overdiagnosis, overtreatment, or adverse psychological responses. In contrast, the VIVA trial [45] showed reduced all-cause mortality but more hospital days for chronic obstructive pulmonary disease and stroke or transient ischaemic attack in the screened group, suggesting that more intensive detection and treatment may simultaneously increase health care use and mediate survival gains. Consistent with this, the increased hospitalization observed in older women in another trial was primarily driven by surgery and gynecology services, possibly reflecting increased therapeutic interventions following earlier diagnosis rather than deterioration in underlying health status [27]. Additionally, a large nationwide cohort from Taiwan found that while depression screening reduced overall psychiatric hospitalization, it was associated with a modestly increased risk of suicide among older adults [67]. This excess risk was concentrated shortly after the health check, leading authors to suggest that newly disclosed severe diagnoses or health concerns in older adults might transiently heighten psychological distress. Taken together, these findings suggest that potential adverse effects, particularly among older or vulnerable populations, should be carefully considered and monitored during implementation of health checks.

This review provides an updated and comprehensive synthesis of the available evidence. We incorporated a wide array of designs, ranging from formal economic evaluations to randomized and observational trials, across multiple countries and healthcare systems. By synthesizing outcomes across the full continuum of mortality, major cardiovascular events, and risk factor control to health care utilization, health behaviors, and patient-reported, psychological, and economic outcomes, this review offers a broader synthesis of the impact of general health checks. We further distinguished findings in general populations from those in older or higher-risk groups. In addition, by incorporating psychological and economic outcomes, which were less extensively evaluated in earlier reviews, this analysis addresses several previously underexplored areas. Finally, the inclusion of several large-scale studies with long follow-up periods also enhances the relevance of the findings to current clinical and public health practice.

This study also has important limitations. First, this review did not include quantitative meta-analysis due to substantial heterogeneity in study design, intervention components, and outcome measures. As a result, effect sizes were not statistically pooled, which may limit the precision of the estimates. The absence of pooled estimates precluded formal assessment of publication bias using funnel plots. Second, many of the randomized trials were conducted decades ago, before the widespread use of statins, contemporary antihypertensive regimens, structured multifactorial cardiovascular risk management, and current cancer screening practices. As a result, the applicability of some findings to current clinical practice may be limited. Third, although randomized trials provide the most reliable evidence on mortality and cardiovascular events, our conclusions for several domains, particularly health behaviors, psychological outcomes, and economic outcomes, also rely on observational studies that are vulnerable to residual confounding, healthy-user effects, and selective participation. Fourth, outcome definitions, measurement methods, and follow-up durations varied across studies; many behavioral, quality-of-life, and psychological outcomes were self-reported, increasing the risk of measurement bias. Fifth, approximately 20% of the included studies were identified through manual screening rather than the electronic search strategy. As noted earlier, several of these were older seminal trials or were indexed under alternative terminology not consistently captured by database indexing systems. Although multiple databases and supplementary reference screening were conducted to enhance comprehensiveness, this proportion may suggest that the electronic search strategy was not fully sensitive. Therefore, the possibility that some eligible studies were not retrieved cannot be entirely excluded. Finally, we included only studies published in English or Chinese. Although comprehensive search strategies were used and unpublished data were included where available, language bias, publication bias, and selective outcome reporting cannot be completely excluded, particularly for smaller studies and outcomes related to psychological harms and costs.

## Conclusions

In this comprehensive systematic review, we found no consistent evidence from randomized trials that general health checks substantially reduce mortality, major cardiovascular events, or hospitalizations. By contrast, general health checks appeared to be associated with earlier detection of chronic conditions, modest improvements in cardiovascular risk factor control, greater uptake of preventive services and pharmacotherapy, and small gains in patient-reported and psychological outcomes, alongside generally favorable short-term economic outcomes. Potential survival benefits appeared more evident among older adults or individuals at elevated cardiovascular risk, whereas universal, low-intensity checks in unselected adults seem to yield limited clinical benefit. Although some newly included studies were conducted in low- and middle-income countries, the evidence base from these settings remains limited. Therefore, the applicability of these findings beyond predominantly Western or high-income settings should be interpreted with caution. A small number of studies also reported potential adverse or ambivalent findings, suggesting that potential unintended consequences should also be monitored. Overall, the findings suggest that a uniform approach to GHCs may not yield consistent benefits across populations. Rather, the effectiveness of GHCs appears to depend on population characteristics, baseline risk, and implementation context. Future strategies may consider more targeted and integrated approaches, particularly for high-risk groups and older adults, while further high-quality research is needed to clarify long-term clinical and economic outcomes.

## Supplementary Information

Supplementary Material 1.

Supplementary Material 2.

Supplementary Material 3.

## Data Availability

All data generated or analyzed during this study are included in this published article and its supplementary information files. Additional data extraction forms are available from the corresponding author upon reasonable request.

## Abbreviations

GHCs: General health checks
MeSH: Medical Subject Heading
RCTs: Randomized controlled trials
NRCTs: Nonrandomized controlled trials
