# Supplementary material for: Effectiveness and cost-effectiveness of general health checks in adults: A systematic review
Source: BMC Public Health. 2026 Jul 8;26:2720. doi: 10.1186/s12889-026-28436-4 (PMC13629029; doi:10.1186/s12889-026-28436-4)
Supplement: Supplementary file 1 [file 12889_2026_28436_MOESM1_ESM.docx]

**eAppendix.** Search strategy

**Medline (via PubMed)**

| Search | Query | Results |
| --- | --- | --- |
| #1 | "physical examination"[MeSH Terms] | 1,439,588 |
| #2 | "annual"[Title] OR "periodic"[Title] OR "yearly"[Title] OR "routine"[Title] | 95,143 |
| #3 | "primary care"[Title/Abstract] OR "primary healthcare"[Title/Abstract] OR "primary health*"[Title/Abstract] OR "general practitioner*"[Title/Abstract] OR "general practice"[Title/Abstract] OR "family doctor*"[Title/Abstract] OR "family practice*"[Title/Abstract] OR "family physician*"[Title/Abstract] | 296,877 |
| #4 | #1 AND (#2 OR #3) | 15,615 |
| #5 | "health check*"[Title] OR "healthcheck*"[Title] OR "health screen*"[Title] OR "healthscreen*"[Title] OR "health evaluat*"[Title] OR "healthevaluat*"[Title] OR "medical check*"[Title] OR "wellness check*"[Title] OR (("annual"[Title] OR "periodic"[Title] OR "regular"[Title] OR "routine"[Title] OR "yearly"[Title]) AND ("check*"[Title] OR "health* exam*"[Title] OR "health* evaluat*"[Title] OR "medical* exam*"[Title] OR "physical* exam*"[Title] OR "wellness check*"[Title] OR "physician visit*"[Title] OR "doctor visit*"[Title] OR "medical*"[Title] OR "physical*"[Title])) OR ("preventive*"[Title] AND ("care check*"[Title] OR "checkup*"[Title] OR "check up*"[Title] OR "exam*"[Title])) | 10,022 |
| #6 | "multiphasic screening"[Title/Abstract] OR "multiphasic test*"[Title/Abstract] OR "multiphasic check*"[Title/Abstract] OR "comprehensive health test"[Title/Abstract] OR "general health screening"[Title/Abstract] OR (("diet"[Title/Abstract] OR "smoking"[Title/Abstract] OR "exercise"[Title/Abstract] OR "lifestyle"[Title/Abstract] OR "weight reduction"[Title/Abstract] OR "physical activity"[Title/Abstract]) AND ("screen*"[Title/Abstract] OR "check*"[Title/Abstract]) AND ("prevention"[Title/Abstract] OR "preventive"[Title/Abstract])) | 11,648 |
| #7 | #4 OR #5 OR #6 | 36,166 |
| #8 | "Primary Health Care"[MeSH Terms] OR "Family Practice"[MeSH Terms] OR "physicians, primary care"[MeSH Terms] OR "General Practice"[MeSH Terms] OR "physicians, family"[MeSH Terms] OR "General Practitioners"[MeSH Terms] OR "outpatient clinics, hospital"[MeSH Terms] OR "Ambulatory Care"[MeSH Terms] OR "Ambulatory Care Facilities"[MeSH Terms] OR "Community Health Services"[MeSH Terms] OR "Community Health Centers"[MeSH Terms] | 708,607 |
| #9 | "primary care"[Title/Abstract] OR "primary healthcare"[Title/Abstract] OR "family practi*"[Title/Abstract] OR "family doctor*"[Title/Abstract] OR "family physician*"[Title/Abstract] OR "general practi*"[Title/Abstract] | 273,470 |
| #10 | #8 OR #9 | 839,203 |
| #11 | "Randomized Controlled Trial"[Publication Type] OR "Controlled Clinical Trial"[Publication Type] OR "Clinical Trials as Topic"[MeSH Terms:noexp] OR "randomized"[Title/Abstract] OR "placebo"[Title/Abstract] OR "randomly"[Title/Abstract] | 1,638,193 |
| #12 | (("Non-Randomized Controlled Trials as Topic"[MeSH Terms] OR "quasi experiment*"[Title/Abstract] OR "quasi experiment*"[Title/Abstract]) AND "quasiexperiment*"[Title/Abstract]) OR "natural experiment*"[Title/Abstract] | 29,199 |
| #13 | "cohort studies"[MeSH Terms:noexp] OR "longitudinal studies"[MeSH Terms:noexp] OR "follow-up studies"[MeSH Terms:noexp] OR "prospective studies"[MeSH Terms:noexp] OR "retrospective studies"[MeSH Terms:noexp] OR "cohort study"[Title/Abstract] OR "longitudinal study"[Title/Abstract] OR "prospective study"[Title/Abstract] OR "retrospective study"[Title/Abstract] | 2,980,206 |
| #14 | "case-control studies"[MeSH Terms:noexp] OR "control groups"[MeSH Terms:noexp] OR "case-control"[Title/Abstract] OR "case-control"[Title/Abstract] OR "control group"[Title/Abstract] OR "control groups"[Title/Abstract] | 993,427 |
| #15 | (#11 OR #12 OR #13 OR #14) NOT ("Animals"[Mesh] NOT "Humans"[Mesh]) | 4,728,404 |
| #16 | #7 AND #10 AND #15 | 5,797 |
| #17 | "Cost-Benefit Analysis"[MeSH Terms] OR "cost effectiveness analysis"[MeSH Terms] OR "cost effectiveness analysis"[MeSH Terms] OR "Cost-Benefit Analysis"[MeSH Terms] OR "economics"[MeSH Terms] | 757,180 |
| #18 | "cost*"[Title/Abstract] OR "economic*"[Title/Abstract] OR "business case"[Title/Abstract] OR "value for money"[Title/Abstract] OR "economic evaluation*"[Title/Abstract] OR "cost effective analysis"[Title/Abstract] OR "cost utility analysis"[Title/Abstract] OR "cost benefit analysis"[Title/Abstract] OR "cost consequence* analysis"[Title/Abstract] OR "cost minimization analysis"[Title/Abstract] OR "return on investment"[Title/Abstract] OR "return to investment"[Title/Abstract] OR "social return on investment"[Title/Abstract] OR "cost effective*"[Title/Abstract] OR "cost utility"[Title/Abstract] OR "cost benefit"[Title/Abstract] OR "efficien*"[Title/Abstract] OR "cost saving*"[Title/Abstract] OR "cost analysis"[Title/Abstract] OR "return on"[Title/Abstract] | 2,625,979 |
| #19 | #17 OR #18 | 3,125,371 |
| #20 | #7 AND #10 AND #19 | 2,037 |

**Embase (via Embase.org)**

| Search | Query | Results |
| --- | --- | --- |
| #1 | 'physical examination'/exp | 384,674 |
| #2 | annual:ti OR periodic:ti OR yearly:ti OR routine:ti | 120,179 |
| #3 | 'primary care':ti,ab,kw OR 'primary healthcare':ti,ab,kw OR 'primary health*':ti,ab,kw OR 'general practitioner*':ti,ab,kw OR 'general practice':ti,ab,kw OR 'family doctor*':ti,ab,kw OR 'family practice*':ti,ab,kw OR 'family physician*':ti,ab,kw | 402,797 |
| #4 | #1 AND (#2 OR #3) | 8,588 |
| #5 | 'health check*':ti OR healthcheck*:ti OR 'health screen*':ti OR healthscreen*:ti OR 'health evaluat*':ti OR healthevaluat*:ti OR 'medical check*':ti OR 'wellness check*':ti OR ((annual:ti OR periodic:ti OR regular:ti OR routine:ti OR yearly:ti) AND (check*:ti OR 'health* exam*':ti OR 'health* evaluat*':ti OR 'medical* exam*':ti OR 'physical* exam*':ti OR 'wellness check*':ti OR 'physician visit*':ti OR 'doctor visit*':ti OR medical*:ti OR physical*:ti)) OR (preventive*:ti AND ('care check*':ti OR checkup*:ti OR 'check up*':ti OR exam*:ti)) | 11,597 |
| #6 | 'multiphasic screening':ti,ab,kw OR 'multiphasic test*':ti,ab,kw OR 'multiphasic check*':ti,ab,kw OR 'comprehensive health test':ti,ab,kw OR 'general health screening':ti,ab,kw OR ((diet:ti,ab,kw OR smoking:ti,ab,kw OR exercise:ti,ab,kw OR lifestyle:ti,ab,kw OR 'weight reduction':ti,ab,kw OR 'physical activity':ti,ab,kw) AND (screen*:ti,ab,kw OR check*:ti,ab,kw) AND (prevention:ti,ab,kw OR preventive:ti,ab,kw)) | 19,162 |
| #7 | #4 OR #5 OR #6 | 38,458 |
| #8 | 'primary health care'/exp OR 'general practice'/exp OR 'general practitioner'/exp OR 'outpatient department'/exp OR 'ambulatory care'/exp OR 'community care'/exp OR 'health center'/exp | 744,450 |
| #9 | 'primary care':ti,ab,kw OR 'primary healthcare':ti,ab,kw OR 'family practi*':ti,ab,kw OR 'family doctor*':ti,ab,kw OR 'family physician*':ti,ab,kw OR 'general practi*':ti,ab,kw | 378,770 |
| #10 | #8 OR #9 | 843,802 |
| #11 | 'randomized controlled trial'/exp OR 'controlled clinical trial'/exp OR 'clinical trial (topic)'/exp OR 'randomized controlled trial':it OR 'controlled clinical trial':it OR 'clinical trial':it OR randomized:ti,ab,kw OR placebo:ti,ab,kw OR randomly:ti,ab,kw | 2,973,850 |
| #12 | 'quasi experimental study'/exp OR 'non-randomized controlled trials':ti,ab,kw OR 'quasi experiment*':ti,ab,kw OR quasiexperiment*:ti,ab,kw OR 'natural experiment*':ti,ab,kw | 42,006 |
| #13 | 'cohort analysis'/exp OR 'longitudinal study'/exp OR 'prospective study'/exp OR 'retrospective study'/exp OR 'cohort study':ti,ab,kw OR 'longitudinal study':ti,ab,kw OR 'prospective study':ti,ab,kw OR 'retrospective study':ti,ab,kw | 3,822,385 |
| #14 | 'case control study'/exp OR 'control group'/exp OR 'case control':ti,ab,kw OR 'control group':ti,ab,kw OR 'control groups':ti,ab,kw | 1,247,638 |
| #15 | (#11 OR #12 OR #13 OR #14) NOT ('animal'/exp NOT 'human'/exp) | 6,729,549 |
| #16 | #7 AND #10 AND #15 | 3,058 |
| **#17** | **#16 AND ([adult]/lim OR [young adult]/lim OR [middle aged]/lim OR [aged]/lim OR [very elderly]/lim)** | **2,133** |
| #18 | 'cost benefit analysis'/exp OR cost*:ti,ab,kw OR economic*:ti,ab,kw OR 'business case':ti,ab,kw OR 'value for money':ti,ab,kw OR 'economic evaluation*':ti,ab,kw OR 'cost effective analysis':ti,ab,kw OR 'cost utility analysis':ti,ab,kw OR 'cost benefit analysis':ti,ab,kw OR 'cost consequence* analysis':ti,ab,kw OR 'cost minimization analysis':ti,ab,kw OR 'return on investment':ti,ab,kw OR 'return to investment':ti,ab,kw OR 'social return on investment':ti,ab,kw OR 'cost effective*':ti,ab,kw OR efficien*:ti,ab,kw OR 'cost saving*':ti,ab,kw OR 'cost analysis':ti,ab,kw OR 'return on':ti,ab,kw | 3,239,701  3,241,679 |
| #19 | #7 AND #10 AND #18 | 1,735 |
| **#20** | **#19 AND ([adult]/lim OR [young adult]/lim OR [middle aged]/lim OR [aged]/lim OR [very elderly]/lim)** | **967** |

**Web of Science**

| Search | Query | Results |
| --- | --- | --- |
| #1 | TS=("physical examination") | 52,377 |
| #2 | TI=(annual OR periodic OR yearly OR routine) | 149,577 |
| #3 | TS=("primary care" OR "primary healthcare" OR "primary health*" OR "general practitioner*" OR "general practice" OR "family doctor*" OR "family practice*" OR "family physician*") | 260,110 |
| #4 | #1 AND (#2 OR #3) | 2,506 |
| #5 | TI=(“health check*” OR healthcheck* OR “health screen*” OR healthscreen* OR “health evaluat*” OR healthevaluat* OR “medical check*” OR “wellness check*” OR ((annual OR periodic OR regular OR routine OR yearly) AND (check* OR “health* exam*” OR “health* evaluat*” OR “medical* exam*” OR “physical* exam*” OR “wellness check*” OR “physician visit*” OR “doctor visit*” OR medical* OR physical*)) OR (preventive* AND (“care check*” OR checkup* OR check-up* OR exam*))) | 7,850 |
| #6 | TS=(“multiphasic screening” OR “multiphasic test*” OR “multiphasic check*” OR “comprehensive health test” OR “general health screening” OR ((diet OR smoking OR exercise OR lifestyle OR “weight reduction” OR “physical activity”) AND (screen* OR check*) AND (prevention OR preventive))) | 14,020 |
| #7 | #4 OR #5 OR #6 | 24,055 |
| #8 | TS=(“primary care” OR “primary healthcare” OR “primary health care” OR “family practi*” OR “family doctor*” OR “family physician*” OR “general practi*” OR “outpatient clinic*” OR "Ambulatory Care" OR "Ambulatory Care Facilities" OR "Community Health Services" OR "Community Health Centers") | 302,611 |
| #9 | TS=("Randomized Controlled Trial" OR “Controlled Clinical Trial” OR "Clinical Trials" OR randomized OR placebo OR randomly) | 1,809,830 |
| #10 | TS=("Non-Randomized Controlled Trials" OR “quasi experiment*” OR quasi-experiment* OR quasiexperiment* OR “natural experiment*”) | 52,576 |
| #11 | TS=(“cohort study” OR “longitudinal study” OR “prospective study” OR “retrospective study” OR “follow-up study”) | 862,285 |
| #12 | TS=(“case control” OR case-control OR “control group” OR “control groups”) | 702,274 |
| #13 | #9 OR #10 OR #11 OR #12 | 3,142,289 |
| **#14** | **#7 AND #8 AND #13** | **1,198** |
| #15 | TS=(cost* OR economic* OR “business case” OR “value for money” OR “economic evaluation*” OR “cost effective analysis” OR “cost utility analysis” OR “cost benefit analysis” OR “cost consequence* analysis” OR “cost minimization analysis” OR “return on investment” OR “return to investment” OR “social return on investment” OR “cost effective*” OR “cost utility” OR “cost benefit” OR efficien* OR “cost saving*” OR “cost analysis” OR “return on”) | 6,512,443 |
| **#16** | **#7 AND #8 AND #15** | **705** |

**Cochrane Library**

| Search | Query | Results |
| --- | --- | --- |
| #1 | MeSH descriptor: [Physical Examination] this term only | 1,116 |
| #2 | (annual OR periodic OR yearly OR routine):ti | 7,168 |
| #3 | (primary care OR primary healthcare OR primary health* OR general practitioner* OR “general practice” OR family doctor* OR family practice* OR family physician*):ti,ab,kw | 285,085 |
| #4 | #1 AND (#2 OR #3) | 186 |
| #5 | (health check* OR healthcheck* OR health screen* OR healthscreen* OR health evaluat* OR healthevaluat* OR medical check* OR wellness check* OR ((annual OR periodic OR regular OR routine OR yearly) AND (check* OR health* exam* OR health* evaluat* OR medical* exam* OR physical* exam* OR wellness check* OR physician visit* OR doctor visit* OR medical* OR physical*)) OR (preventive* AND (care check* OR checkup* OR check-up* OR exam*))):ti | 4,840 |
| #6 | (multiphasic screening OR multiphasic test* OR multiphasic check* OR “comprehensive health test” OR “general health screening” OR ((diet OR smoking OR exercise OR lifestyle OR weight reduction OR physical activity) AND (screen* OR check*) AND (prevention OR preventive))):ti,ab,kw | 4,261 |
| #7 | #4 OR #5 OR #6 | 9,141 |
| #8 | MeSH descriptor: [Primary Health Care] this term only | 6,631 |
| #9 | MeSH descriptor: [Family Practice] this term only | 2,342 |
| #10 | MeSH descriptor: [Physicians, Primary Care] this term only | 239 |
| #11 | MeSH descriptor: [General Practice] this term only | 787 |
| #12 | MeSH descriptor: [Physicians, Family] this term only | 541 |
| #13 | MeSH descriptor: [General Practitioners] this term only | 602 |
| #14 | MeSH descriptor: [Outpatient Clinics, Hospital] this term only | **633** |
| #15 | MeSH descriptor: [Ambulatory Care] this term only | 3,850 |
| #16 | MeSH descriptor: [Ambulatory Care Facilities] this term only | **798** |
| #17 | MeSH descriptor: [Community Health Services] this term only | 1,380 |
| #18 | MeSH descriptor: [Community Health Centers] this term only | 298 |
| #19 | (primary care OR primary healthcare OR family practi* OR family doctor* OR family physician* OR general practi*):ti,ab,kw | 166,481 |
| #20 | #8 OR #9 OR #10 OR #11 OR #12 OR #13 OR #14 OR #15 OR #16 OR #17 OR #18 OR #19 | 171,006 |
| #21 | ("Randomized Controlled Trials" OR "Clinical Trials" OR "randomized controlled trials" OR RCT OR "randomised control trials" OR "clinical controlled trial" OR "clinical trial" OR randomized OR placebo OR randomly):ti,ab,kw | 1,553,624 |
| #22 | ("Quasi-Experimental Studies" OR "Experimental Studies" OR "Nonrandomized Trials" OR quasi experiment* OR quasi-experiment* OR quasiexperiment* OR natural experiment*):ti,ab,kw | 16,870 |
| #23 | ("Prospective Studies" OR "Retrospective Design" OR "Retrospective Panel Studies" OR “cohort study” OR “longitudinal study” OR “prospective study” OR “retrospective study” OR “follow-up study”):ti,ab,kw | 218,780 |
| #24 | ("Case Control Studies" OR "Control Group" OR “case control” OR case-control OR “control group” OR “control groups”):ti,ab,kw | 325,468 |
| #25 | (#21 OR #22 OR #23 OR #24) | 1,656,039 |
| **#26** | **#7 AND #20 AND #25** | **2651** |
| #27 | MeSH descriptor: [Cost-Benefit Analysis] this term only | 11,547 |
| #28 | MeSH descriptor: [Cost-Effectiveness Analysis] this term only es | 235 |
| #29 | (cost* OR economic* OR “business case” OR “value for money” OR economic evaluation* OR “cost effective analysis” OR “cost utility analysis” OR “cost benefit analysis” OR cost consequence* analysis OR “cost minimization analysis” OR “return on investment” OR “return to investment” OR “social return on investment” OR cost effective* OR “cost utility” OR “cost benefit” OR efficien* OR cost saving* OR “cost analysis” OR “return on”):ti,ab,kw | 174,877 |
| #30 | #27 OR #28 OR #29 | 174,877 |
| **#31** | **#7 AND #20 AND #30** | **1,006** |

**CINAHL (via EBSCO)**

| Search | Query | Results |
| --- | --- | --- |
| #1 | MH "Physical Examination" | 32,459 |
| #2 | TI (annual OR periodic OR yearly OR routine) | 71,057 |
| #3 | XB (“primary care” OR “primary healthcare” OR “primary health*” OR “general practitioner*” OR “general practice” OR “family doctor*” OR “family practice*” OR “family physician*”) | 134,849 |
| #4 | #1 AND (#2 OR #3) | 1,688 |
| #5 | TI (“health check*” OR healthcheck* OR “health screen*” OR healthscreen* OR “health evaluat*” OR healthevaluat* OR “medical check*” OR “wellness check*” OR ((annual OR periodic OR regular OR routine OR yearly) AND (check* OR “health* exam*” OR “health* evaluat*” OR “medical* exam*” OR “physical* exam*” OR “wellness check*” OR “physician visit*” OR “doctor visit*” OR medical* OR physical*)) OR (preventive* AND (“care check*” OR checkup* OR check-up* OR exam*))) | 6,533 |
| #6 | XB (“multiphasic screening” OR “multiphasic test*” OR “multiphasic check*” OR “comprehensive health test” OR “general health screening” OR ((diet OR smoking OR exercise OR lifestyle OR “weight reduction” OR “physical activity”) AND (screen* OR check*) AND (prevention OR preventive))) | 3,900 |
| #7 | #4 OR #5 OR #6 | 11,826 |
| #8 | MH "Primary Health Care" OR MH "Family Practice" OR MH "Physicians, Family" OR MH "Outpatients" OR MH "Ambulatory Care" OR MH "Ambulatory Care Facilities" OR MH "Community Health Services" OR MH "Community Health Centers" | 209,902 |
| #9 | XB (“primary care” OR “primary healthcare” OR “family practi*” OR “family doctor*” OR “family physician*” OR “general practi*”) | 124,414 |
| #10 | #8 OR #9 | 266,892 |
| #11 | (MH "Randomized Controlled Trials" OR MH "Clinical Trials") OR PT ("randomized controlled trials" OR RCT OR "randomised control trials" OR "clinical controlled trial" OR "clinical trial") OR XB (randomized OR placebo OR randomly) | 555,947 |
| #12 | (MH "Quasi-Experimental Studies" OR MH "Experimental Studies" OR MH "Nonrandomized Trials") OR XB (“quasi experiment*” OR quasi-experiment* OR quasiexperiment* OR “natural experiment*”) | 58,602 |
| #13 | (MH "Prospective Studies" OR MH "Retrospective Design" OR MH "Retrospective Panel Studies") OR XB (“cohort study” OR “longitudinal study” OR “prospective study” OR “retrospective study” OR “follow-up study”) | 884,531 |
| #14 | (MH "Case Control Studies" OR MH "Control Group") OR XB (“case control” OR case-control OR “control group” OR “control groups”) | 237,503 |
| #15 | (#11 OR #12 OR #13 OR #14) NOT (MH "Animals" NOT MH "Human") | 1,494,421 |
| **#16** | **#7 AND #10 AND #15** | **515** |
| #17 | (MH "Cost Benefit Analysis" OR MH "Cost Effectiveness Analysis" OR MH "Cost Savings") OR XB (cost* OR economic* OR “business case” OR “value for money” OR “economic evaluation*” OR “cost effective analysis” OR “cost utility analysis” OR “cost benefit analysis” OR “cost consequence* analysis” OR “cost minimization analysis” OR “return on investment” OR “return to investment” OR “social return on investment” OR “cost effective*” OR “cost utility” OR “cost benefit” OR efficien* OR “cost saving*” OR “cost analysis” OR “return on”) | 426,102 |
| **#18** | **#7 AND #10 AND #17** | **285** |

**万方（Wanfang）**

| **序号** | **检索策略** | **数量** |
| --- | --- | --- |
| #1 | 主题:(体格检查 OR 多相筛查 OR 普查 OR 普检 OR 筛查 OR 健康筛查 OR 疾病筛查 OR 定期体检 OR 年度体检 OR 常规体检 OR 预防性体检 OR 入职体检 OR 健康检查 OR 健康体检 OR 疾病筛查 OR 早期筛查 OR 早期诊断) | 784,661 |
| #2 | 主题:(初级卫生保健 OR 初级保健 OR 基层卫生服务 OR 基层医疗 OR 家庭医生 OR 家庭医疗保健服务 OR 社区医生 OR 基层医生 OR 全科医生 OR 门诊医疗 OR 门诊诊疗 OR 门诊服务 OR 社区卫生服务 OR 社区卫生中心 OR 社区诊所 OR 社区医院) | 193,630 |
| #3 | 主题:(随机对照 OR 随机 OR 临床试验 OR RCT OR 随机研究 OR 干预研究 OR 类试验研究 OR 非随机对照 OR 准试验研究 OR 对照研究 OR 对照组 OR 病例对照 OR 队列研究 OR 纵向研究 OR 前瞻性研究 OR 回顾性研究 OR 应用效果 OR 干预效果 OR 效果 OR 疗效) | 13,321,035 |
| **#4** | **#1 AND #2 AND #3** | **6,625** |
| #5 | 主题:(成本 OR 成本分析 OR 成本效益 OR 成本效用 OR 成本效果 OR 卫生经济学 OR 经济效益 OR 经济效用 OR 经济评价 OR 经济学评价 OR 费用效益 OR 效益-成本分析 OR 投资回报) | 3,592,677 |
| **#6** | **#1 AND #2 AND #5** | **1,040** |

**知网（CNKI）**

| **序号** | **检索策略** | **数量** |
| --- | --- | --- |
| #1 | TKA=(体格检查 + 多相筛查 + 普查 + 普检 + 筛查 + 健康筛查 + 疾病筛查 + 定期体检 + 年度体检 + 常规体检 + 预防性体检 + 入职体检 + 健康检查 + 健康  体检 + 疾病筛查 + 早期筛查 + 早期诊断) | 606,443 |
| #2 | TKA=(初级卫生保健 + 初级保健 + 基层卫生服务 + 基层医疗 + 家庭医生 + 家庭医疗保健服务 + 社区医生 + 基层医生 + 全科医生 + 门诊医疗 + 门诊诊疗 + 门诊服务 + 社区卫生服务 + 社区卫生中心 + 社区诊所 + 社区医院) | 110,516 |
| #3 | TKA=(随机对照 + 随机 + 临床试验 + RCT + 随机研究 + 干预研究 + 类试验研究 + 非随机对照 + 准试验研究 + 对照研究 + 对照组 + 病例对照 + 队列研究 + 纵向研究 + 前瞻性研究 + 回顾性研究 + 应用效果 + 干预效果 + 效果 + 疗效) | 11,355,958 |
| **#4** | **#1 AND #2 AND #3** | **2,435** |
| #5 | TKA=(成本 + 成本分析 + 成本效益 + 成本效用 + 成本效果 + 卫生经济学 + 经济效益 + 经济效用 + 经济评价 + 经济学评价 + 费用效益 + 效益-成本分析 + 投资回报) | 3,897,654 |
| **#6** | **#1 AND #2 AND #5** | **581** |

**中国生物医学文献服务系统（SinoMed）**

| **序号** | **检索策略** | **数量** |
| --- | --- | --- |
| #1 | ("体格检查"[加权:扩展] OR "多相筛查"[加权:扩展] OR "普查"[加权:扩展]) | 153,677 |
| #2 | ("体格检查"[核心字段:智能] OR "多相筛查"[核心字段:智能] OR "普查"[核心字段:智能] OR "普检"[核心字段:智能] OR "筛查"[核心字段:智能] OR "健康筛查"[核心字段:智能] OR "疾病筛查"[核心字段:智能] OR "定期体检"[核心字段:智能] OR "年度体检"[核心字段:智能] OR "常规体检"[核心字段:智能] OR "预防性体检"[核心字段:智能] OR "入职体检"[核心字段:智能] OR "健康检查"[核心字段:智能] OR "健康体检"[核心字段:智能] OR "疾病筛查"[核心字段:智能] OR "早期筛查"[核心字段:智能] OR "早期诊断"[核心字段:智能]) | 619,990 |
| #3 | #1 OR #2 | 619,990 |
| #4 | ("初级卫生保健"[加权:扩展] OR "家庭医学"[加权:扩展] OR "家庭医疗保健服务"[加权:扩展] OR "医生, 初级保健"[加权:扩展] OR "全科医生"[加权:扩展] OR "医师, 家庭"[加权:扩展] OR "门诊部, 医院"[加权:扩展] OR "门诊服务"[加权:扩展] OR "社区卫生服务"[加权:扩展] OR "社区卫生中心"[加权:扩展]) | 136,360 |
| #5 | ("初级卫生保健"[核心字段:智能] OR "初级保健"[核心字段:智能] OR "基层卫生服务"[核心字段:智能] OR "基层医疗"[核心字段:智能] OR "家庭医生"[核心字段:智能] OR "家庭医疗保健服务"[核心字段:智能] OR "社区医生"[核心字段:智能] OR "基层医生"[核心字段:智能] OR "全科医生"[核心字段:智能] OR "门诊医疗"[核心字段:智能] OR "门诊诊疗"[核心字段:智能] OR "门诊服务"[核心字段:智能] OR "社区卫生服务"[核心字段:智能] OR "社区卫生中心"[核心字段:智能] OR "社区诊所"[核心字段:智能] OR "社区医院"[核心字段:智能]) | 190,965 |
| #6 | #4 OR #5 | 193,049 |
| #7 | ("随机对照试验"[加权:扩展] OR "临床试验"[加权:扩展] OR "临床对照试验"[加权:扩展] OR "病例对照研究"[加权:扩展] OR "队列研究"[加权:扩展] OR "纵向研究"[加权:扩展] OR "回顾性研究"[加权:扩展]) | 11,189 |
| #8 | ("随机对照"[核心字段:智能] OR "随机"[核心字段:智能] OR "临床试验"[核心字段:智能] OR "RCT"[核心字段:智能] OR "随机研究"[核心字段:智能] OR "干预研究"[核心字段:智能] OR "类试验研究"[核心字段:智能] OR "非随机对照"[核心字段:智能] OR "准试验研究"[核心字段:智能] OR "对照研究"[核心字段:智能] OR "对照组"[核心字段:智能] OR "病例对照"[核心字段:智能] OR "队列研究"[核心字段:智能] OR "纵向研究"[核心字段:智能] OR "前瞻性研究"[核心字段:智能] OR "回顾性研究"[核心字段:智能] OR "应用效果"[核心字段:智能] OR "干预效果"[核心字段:智能] OR "效果"[核心字段:智能] OR "疗效"[核心字段:智能]) | 2,119,044 |
| #9 | #7 OR #8 | 2,119,044 |
| **#10** | **#3 AND #6 AND #9** | **2,493** |
| #11 | ( "成本及成本分析"[加权:扩展] OR "费用效益分析"[加权:扩展]) | 8,113 |
| #12 | ( "成本"[核心字段:智能] OR "成本分析"[核心字段:智能] OR "成本效益"[核心字段:智能] OR "成本效用"[核心字段:智能] OR "成本效果"[核心字段:智能] OR "卫生经济学"[核心字段:智能] OR "经济效益"[核心字段:智能] OR "经济效用"[核心字段:智能] OR "经济评价"[核心字段:智能] OR "经济学评价"[核心字段:智能] OR "费用效益"[核心字段:智能] OR "效益-成本分析"[核心字段:智能] OR "投资回报"[核心字段:智能]) | 63,508 |
| #13 | #11 OR #12 | 63,508 |
| **#14** | **#3 AND #6 AND #13** | **92** |
