# Supplementary material for: Effectiveness and cost-effectiveness of general health checks in adults: A systematic review
Source: BMC Public Health. 2026 Jul 8;26:2720. doi: 10.1186/s12889-026-28436-4 (PMC13629029; doi:10.1186/s12889-026-28436-4)
Supplement: Supplementary file 2 [file 12889_2026_28436_MOESM2_ESM.docx]

**eTable 1.** Summary of tools and scoring rules used for risk-of-bias and quality assessment

| **Study design** | **Tool** | **Main domains / items assessed** | **Scoring of domains / items** | **Overall judgement** |
| --- | --- | --- | --- | --- |
| Randomized controlled trials (RCTs) | Cochrane Risk of Bias tool for randomized trials (RoB 2.0)^[1]^ | 5 domains: (1) randomization process; (2) deviations from intended interventions; (3) missing outcome data; (4) measurement of outcomes; (5) selection of the reported result. | Each domain rated as **“low risk”**, **“some concerns”**, or **“high risk”** of bias. | A comprehensive overall risk-of-bias judgement combining domain ratings, classified as **low risk**, **some concerns**, or **high risk** of bias. |
| Non-randomized controlled trials (nRCTs) | Risk Of Bias In Non-randomized Studies of Interventions (ROBINS-I-V2)^[2,3]^ | 7 domains: (1) confounding bias; (2) selection bias; (3) misclassification of interventions; (4) deviations from intended interventions; (5) measurement bias in outcome assessment; (6) bias due to missing data; (7) selective reporting of results. | Each domain judged as **low**, **moderate**, **serious**, or **critical** risk of bias. | Overall risk-of-bias judgement based on all domains, classified as **low**, **moderate**, **serious**, or **critical** risk of bias. |
| Cohort studies | Newcastle–Ottawa Scale (NOS)^[4]^ | 3 domains: (1) selection of study groups (0–4 stars); (2) comparability of groups (0–2 stars); (3) ascertainment of outcome (0–3 stars). | Each item contributes “stars”; total score ranges from 0 to 9 stars. | **High quality**: 7–9 stars; **moderate quality**: 4–6 stars; **low quality**: 0–3 stars.^[4]^ |
| Cross-sectional studies | Joanna Briggs Institute critical appraisal checklist (JBI)^[5]^ | 8 reporting items: clarity of sample inclusion criteria; detailed description of subjects and settings; validity and reliability of exposure and outcome measurements; identification and management of confounding variables, etc.^[6]^ | Each item rated “yes”, “no”, “unclear”, or “not applicable”. For scoring: **yes = 1**, “no”/“unclear”/“not applicable” = 0. Percentage score = (sum of “yes” / total items) × 100%. | **High quality**: >80%; **moderate quality**: 60–80%; **low quality**: <60%.^[7]^ |
| Economic evaluations | Drummond's checklist, in Methods for the Economic Evaluation of Health Care Programmes^[8]^ | 10 domains comprising 33 methodological criteria assessing the design, conduct, and analytical rigour of economic evaluations. | Each criterion was rated as “fulfilled” =1 or “not fulfilled”/ “unclear” =0. Percentage score = (sum of “fulfilled” / 33) × 100%. | High **quality**: ≥85%; moderate **quality**: 61–84%; low **quality**: ≤60%.^[9]^ |
| Mixed-methods studies | Mixed Methods Appraisal Tool, 2018 version (MMAT 2018)^[10]^ | Tool divided into 3 categories by design: mixed methods, qualitative, and quantitative (randomised controlled, nonrandomised, descriptive). | For each applicable item: “yes”, “no”, or “can’t tell”. For scoring: **yes = 1**, “no”/“can’t tell” = 0. Percentage score = (sum of “yes” / number of applicable items) × 100%. | **High quality**: 100%; **considerable quality**: 75–<100%; **moderate quality**: 50–<75%; **low quality**: 25–<50%; very low quality: <25%.^[11]^ |

**References**

[1] Sterne JAC, Savović J, Page MJ, et al. RoB 2: a revised tool for assessing risk of bias in randomised trials. BMJ. 2019;366:l4898. Published 2019 Aug 28. doi:10.1136/bmj.l4898
[2] Higgins JP, Altman DG, Gøtzsche PC, et al. The Cochrane Collaboration's tool for assessing risk of bias in randomised trials. BMJ. 2011;343:d5928. Published 2011 Oct 18. doi:10.1136/bmj.d5928
[3] Sterne JA, Hernán MA, Reeves BC, et al. ROBINS-I: a tool for assessing risk of bias in non-randomised studies of interventions. BMJ. 2016;355:i4919. Published 2016 Oct 12. doi:10.1136/bmj.i4919
[4] Wells G, Shea B, O’Connell D, et al. The Newcastle-Ottawa Scale (NOS) for assessing the quality of nonrandomised studies in meta-analyses. Ottawa Hospital Research Institute website. Published 2013. Accessed August 10, 2025. Available at: <http://www.ohri.ca/programs/clinical_epidemiology/oxford.asp>
[5] Joanna Briggs Institute. Joanna Briggs Institute Critical Appraisal Tools. Joanna Briggs Institute website. Published 2025. Accessed August 12, 2025. Available at: <https://jbi.global/critical-appraisal-tools>
[6] Moola S, Munn Z, Tufanaru C, et al. Systematic reviews of etiology and risk. In: Aromataris E, Munn Z, eds. JBI Manual for Evidence Synthesis. 2020. Accessed August 12, 2025. Available at: <https://synthesismanual.jbi.global>
[7] Fauziah H.F.N., Rochmawati E., Padela A. A systematic review and meta-analysis of sleep hygiene implementation and its effect on sleep quality and fatigue in patients undergoing hemodialysis. J Ners. 2024;19(4):520–530. doi: 10.20473/jn.v19i4.63368.
[8] Drummond MF, Sculpher MJ, Claxton K, et al. Methods for the Economic Evaluation of Health Care Programmes. 4th ed. Oxford University Press; 2015.
[9] Al Miaraj A, Tomini SM, Ward N, Catena R, et al. Economic evaluations of outpatient stroke rehabilitation models: a systematic review of evidence, methods, and gaps. EClinicalMedicine. 2026;94:103875. doi:10.1016/j.eclinm.2026.103875.
[10] Hong QN, Fàbregues S, Bartlett G, et al. The Mixed Methods Appraisal Tool (MMAT) version 2018 for information professionals and researchers. Educ Inf. 2018;34(4):285-291. doi:10.3233/EFI-180221
[11] Hedima EW, Okoro RN. Primary health care roles of community pharmacists in low- and middle-income countries: a mixed methods systematic review. BMC Health Serv Res. 2025;25(1):1269. Published 2025 Oct 1. doi:10.1186/s12913-025-13387-0
